# Supplementary material for: MetaRibo-Seq measures translation in microbiomes
Source: Nat Commun. 2020 Jun 29;11:3268. doi: 10.1038/s41467-020-17081-z (PMC7324362; doi:10.1038/s41467-020-17081-z)
Supplement: Supplementary file 10 — Supplementary Data 7 [file 41467_2020_17081_MOESM10_ESM.zip › File2/Confidence_High_Taxonomy/347363_out.krona.html]

Javascript must be enabled to view this page.

members
magnitude
magnitudeUnassigned
count
unassigned
taxon
rank

347363\_out

18

18
2
superkingdom

976
phylum
18

18
class
200643

171549
order
18

18
family
815

18
816
genus


SRS018541\_contig\_number\_12690SRS019161\_contig\_number\_11991SRS022609\_contig\_number\_19643SRS046717\_contig\_number\_2475SRS1041112\_contig\_number\_3235SRS104636\_contig\_number\_21576SRS143523\_contig\_number\_25236SRS149784\_contig\_number\_11437SRS893342\_contig\_number\_10422
821
species
9


SRS045826\_contig\_number\_3596
47678
species
1


SRS014923\_contig\_number\_43943SRS023346\_contig\_number\_3034SRS057717\_contig\_number\_2311SRS143598\_contig\_number\_14625
species
2292943
4

1
species
357276

SRS140645\_contig\_number\_157

3
2292935
species

SRS1055043\_contig\_number\_25425SRS1055067\_contig\_number\_14233SRS147653\_contig\_number\_5783
